# Visual Attention and Motion Visibility Modulate Motor Resonance during Observation of Human Walking in Different Manners

**DOI:** 10.3390/brainsci11060679

**Published:** 2021-05-22

**Authors:** Tomotaka Ito, Masanori Kamiue, Tomonori Kihara, Yuta Ishimaru, Daisuke Kimura, Akio Tsubahara

**Affiliations:** 1 Department of Physical Therapy, Faculty of Rehabilitation, Kawasaki University of Medical Welfare, Kurashiki-City, Okayama 701-0193, Japan; kimura.d@mw.kawasaki-m.ac.jp (D.K.); atsuba@mw.kawasaki-m.ac.jp (A.T.); 2 Doctoral Program in Rehabilitation, Graduate School of Health Science and Technology, Kawasaki University of Medical Welfare, Kurashiki-City, Okayama 701-0193, Japan; p_reticulata_0229@yahoo.co.jp; 3 Department of Rehabilitation, Kasaoka Daiichi Hospital, Kasaoka-City, Okayama 714-0043, Japan; iekwq720@yahoo.co.jp; 4 Department of Rehabilitation, Kurashiki Sweet Hospital, Kurashiki-City, Okayama 710-0016, Japan; tennisboy.0721yuta@outlook.com

**Keywords:** action observation, motor resonance, gait, attention, transcranial magnetic stimulation

## Abstract

To advance our knowledge on the motor system during cyclic gait observation, we aimed to explore the effects of gaze fixation on corticospinal excitability evaluated by single-pulse transcranial magnetic stimulation (TMS). Fourteen healthy adult volunteers watched a video of a demonstrator walking on a treadmill under three different conditions: (1) observing the right lower limb, (2) observing the right ankle joint, and (3) observing the right lower limb on a video focused on the area below the knee. In each condition, motor-evoked potentials elicited by TMS in the tibialis anterior (TA) muscle were measured synchronously with the demonstrator’s initial contact and toe-off points. Directing visual attention to the ankle joint and focusing on its movements caused corticospinal facilitation in the TA muscle compared with watching the video without any visual fixation. In addition, phase-dependent differences in corticospinal excitability between the initial contact and toe-off points were only detected when the visibility range was restricted to below the knee. Our findings indicated that motor resonance during cyclic gait observation is modulated by visual attention and motion visibility in different activation manners.

## 1. Introduction

Over the past several decades, a large number of studies using brain imaging and neurophysiological techniques, such as transcranial magnetic stimulation (TMS), have clarified the brain regions [1,2] and properties of the motor system [3] during the observation of actions conducted by others. Action observation is used as an alternative or adjunct to the physical rehabilitation approach and is clinically shown to promote motor (re)learning for the recovery of motor functions [4,5,6,7,8,9]. As several recent meta-analyses have reported that action observation therapy (AOT) is effective in restoring upper limb function [6,7,8] and walking ability [7,9] in patients with stroke, AOT appears to be a beneficial rehabilitation approach for those with upper or lower limb disorders.

The mirror neuron system is now well-accepted as the neural basis of AOT [10], and action observation is widely known to activate similar, but not identical, neural structures associated with the physical execution of the same action in observers [2]. To date, several studies have focused on the neural activities elicited by the observation of movements of the upper limbs. However, to our knowledge, only one study has assessed walking as an experimental task and directly reported the observation/execution matching of brain regions concerning walking using functional magnetic resonance imaging methods [11].

While brain imaging can identify the activation of motor cortical areas in response to movement observation, single-pulse TMS can also reveal the cortical motor resonance to the observed movement. Motor-evoked potentials (MEPs) induced by TMS during action observation are reported to be closely coupled to the muscles involved in the action execution [12,13,14,15,16,17,18,19,20,21,22] and the temporal pattern of the observed action [14,15,22,23,24], which are remarkable and meaningful findings elucidated by TMS studies. Regarding the motor system activity during mere cyclic gait observation, prior TMS studies have demonstrated that the motor resonance to the observed whole-body gait movement was enhanced in the crural flexor and extensor muscles throughout the step cycle [25,26], which is incompatible with the kinematic feature of actual walking in terms of the time course of muscle activity. Although AOT is often used to improve walking ability in clinical settings [4,7,9], the motor cortical activity while observing human bipedal walking is not well-known; for example, it is unknown whether this curious motor resonance during gait observation replicates the original time course of the motor cortical activity during gait execution, or whether it is interfered with by factors affecting the inner automatic motor response process of the observed gait in a top-down modulation manner [27].

Several recent TMS studies have focused on the substantial aspects of observation behavior, that is, what observers look at and fixate their eyes on [28,29,30], and proved that directing visual attention to an acting effector [31] or a task-related object [29] augments the corticospinal excitability compared with a free-viewing condition without gaze fixation. As human bipedal walking is a complex and coordinated multi-joint movement, visual attention or gaze fixation during gait observation seems to be a plausible factor that regulates the observer’s own motor system.

Thus, we aimed to elucidate the effect of directing visual attention to a specific region of the body while watching a video of a walker’s whole-body movement. For this purpose, verbal instructions were given to control the observers’ visual attention (free viewing vs. gaze fixation). Moreover, using an adjusted visibility-range of the gait video (normal vs. restricted visibility), we aimed to examine whether motor resonance during gait observation would be elicited in a gait-specific manner that differs from a simple joint movement.

## 2. Materials and Methods

### 2.1. Participants

Fourteen healthy volunteers (sex, seven men and seven women) aged 20–22 years (mean age, 20.6 ± 0.6 years) without history of neurological or psychiatric disorders participated in the study. As our previous research had explored the effect of visual experience on motor cortical activity during gait observation [26], the participants who had formal experience with gait observation in clinical education were excluded from this study. Prior to conducting the experiments, we confirmed that all participants were right-handed using the Edinburgh Handedness Inventory [32].

### 2.2. Electromyography (EMG)

Electromyographic (EMG) responses to TMS were recorded from the right tibialis anterior (TA) muscle using disposable Ag/AgCl surface electrodes with a 10-mm diameter (Blue Sensor P-00-S; Ambu Co., Ballerup, Denmark). Bipolar electrode pairs were attached longitudinally to the muscle belly at an inter-electrode distance of 20 mm. A reference electrode was placed over the right lateral malleolus. To reduce skin impedance, the skin was abraded with a skin preparation gel (Skin Pure; Nihon Kohden Co., Tokyo, Japan) and, then, cleaned with alcohol before placing the electrodes. The EMG signals were amplified using a bio-amplifier (BA1008; TEAC Co., Tokyo, Japan) and stored on a personal computer for offline analysis. The sensitivity, time constant, and high cut-off filter of the amplifier were set at 200 μV/0.5 V, 0.01 s, and 3 kHz, respectively.

### 2.3. TMS Procedure

MEPs elicited by single-pulse TMS were measured as surrogates of corticospinal excitability. TMS was delivered using a Magstim 200^2^ magnetic stimulator (Magstim Co., Dyfed, UK) connected to a double-cone coil with an outer diameter of 110 mm. After identification of the vertex (Cz) according to the international 10–20 electroencephalogram system [33] and before the initial MEP measurement, the coil was moved over the scalp to find the optimal coil position (i.e., hot spot) that evoked the largest MEP amplitudes in the right TA muscle and was oriented so that the induced electric current in the brain would flow in the posterior–anterior direction. To ensure that the same location was stimulated during individual MEP measurements, the hot spot was marked on a tight-fitting swim cap, which was fixed to the skin with surgical tape. The resting motor threshold was defined as the minimum stimulus intensity necessary to induce an MEP with a peak-to-peak amplitude of 100 μV for at least five out of 10 consecutive stimuli. The experimental stimulus intensity was set at 120% of the resting motor threshold. Stimulus intensity was expressed as the percentage of the maximum stimulator output.

### 2.4. Stimulation Protocol

**Figure 1:**
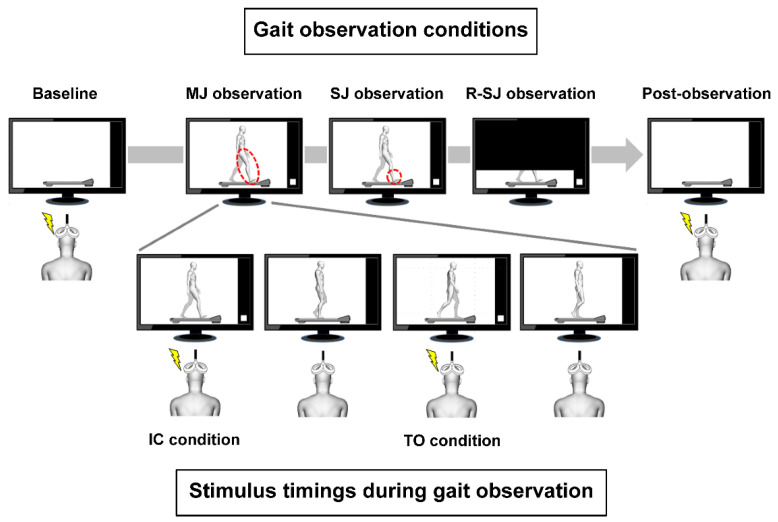
Stimulus timings during gait observation and experimental conditions. The walker’s right and left legs are presented in white and gray color, respectively. The white squares in the monitors were used as trigger signals for magnetic stimulation. The transcranial magnetic stimulation was delivered to the observer at two different points of the gait cycle: when the walker’s right foot contacted the treadmill (initial contact (IC) condition) and when the right foot rose from the treadmill (toe-off (TO) condition). MEP measurements were conducted prior to and following gait observation (baseline and post-observation conditions, respectively), and repeated during the three types of gait observation conditions. The observers were asked to closely observe the walker’s right lower limb movements in the multiple-joint (MJ) observation condition, the walker’s right ankle joint movements in the single-joint (SJ) condition, and the walker’s right lower limb projected only below the knee joints in the restricted single-joint (R-SJ) condition. The red dotted lines indicate the body parts observed in each condition.

Prior to the experiment, we recorded the demonstrator’s gait video from the right sagittal plane. Subsequently, we edited the video using video editing software and marked two points of the gait cycle with a bright spot (white square) to use them as trigger signals for TMS (Figure 1). During a gait cycle, the TA muscle activity begins at the transition phase from stance to swing (pre-swing) and ceases when the foot falls flat on the ground (loading response) [34]. Therefore, to examine the excitability changes in the corticospinal tract to the TA, we selected initial contact (IC) and toe-off (TO) points, as these two timings during a gait cycle represent the distinctive kinematic features in the period of the TA muscle activity (Figure 1). A photosensor was attached to the monitor and used to detect brightness changes in video frames because of the aforementioned bright spots. The timing of the stimuli was strictly synchronized with the demonstrator’s gait cycle using the photosensor. Stimulus parameters, such as the number and order of stimuli, were randomly determined in advance when editing the gait video clips. MEP measurements were obtained before and after gait observation (baseline and post-observation conditions, respectively) with a 150-s rest time between each measurement, and repeated during the three types of gait observation conditions. Eight MEPs were elicited by TMS at baseline, post-observation, and gait observation conditions with an inter-stimulus interval of ≥8 s. In each observation condition, eight stimuli were delivered at the IC and TO points of the demonstrator’s gait cycle (IC and TO stimulus conditions, respectively) in a pseudorandom order. Therefore, a total of 64 MEPs were recorded, including 8, 16, and 8 MEPs in the baseline trial, in each gait observation trial, and in the post-observation trial, respectively. To avoid difficulties in maintaining concentration while observing constant gait movements for a long period, the time length of each edited gait video was set at 2 min and 19 s.

### 2.5. Experimental Conditions

The participants (observers) sat on a comfortable chair with a backrest with their feet not touching the ground, and watched a video on a 27-inch monitor (FlexScan EV2750; EIZO Co., Ishikawa, Japan) located at a distance of 1 m. In the video, a demonstrator walked on a treadmill at a speed of 2 km/h according to a tempo of 60 beats per min produced by a metronome. During the MEP measurements, the observers were instructed to relax their body completely and avoid making any body movement or inducing any muscle contraction. In the baseline and post-observation conditions, the observers were asked to look at a still picture of the treadmill without the walker. The following three conditions were used for gait observation in a pseudorandom order (Figure 1):

(1) In the observation condition of multiple-joint movements (MJ observation), the observers were instructed to closely observe the movements of the walker’s right lower limb while they were watching the monitor projecting the complete image of the demonstrator’s gait.
(2) In the observation condition of single-joint movements (SJ observation), to elucidate the effect of visual attention on the motor system, the observers were instructed to closely observe the movements of the walker’s right ankle joint while they were watching the monitor projecting the complete image of the demonstrator’s gait.
(3) In the observation condition of restricted single-joint movements (R-SJ observation), to elucidate the effect of motion visibility on the motor system, the observers were instructed to closely observe the movements of the walker’s right lower limb while they were watching the monitor projecting the same video, as aforementioned, which was zoomed to include only the area below the knee joints.

In this study, verbal instructions were used to control gaze fixation points or direct visual attention to the demonstrator’s body parts during gait observation in each experimental condition. To further clarify the effect of the observation site on corticospinal excitability, we set the SJ and R-SJ conditions as observation conditions. We hypothesized that if the observers could adhere to individual verbal instructions, the result of MEP changes in the R-SJ condition would resemble that in the SJ condition.

### 2.6. Assessment of Degree of Attention

The visual analog scale (VAS) score has been widely utilized for the subjective assessment of the degree of pain [35,36] and vividness of motor imagery [37]. In the current study, the VAS score was used to clarify the individual degree of attention immediately after each condition of gait observation. The participants placed a mark on a 100-mm horizontal line to show to what extent they could pay attention to each observational video according to the instructions. The left edge of the line was labeled “0 = none at all”, and the right edge was labeled “10 = very high attention”.

### 2.7. Data and Statistical Analyses

**Figure 2:**
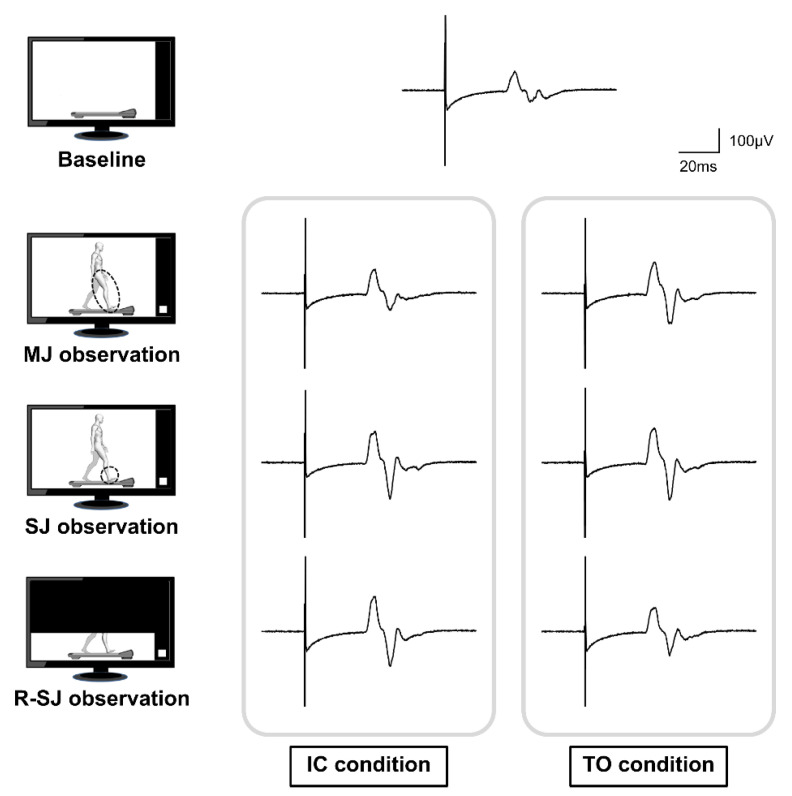
Representative waveforms of the averaged motor evoked potentials (MEPs) obtained from a single participant during the baseline, multiple-joint (MJ), single-joint (SJ), and restricted single-joint (R-SJ) observation conditions. The four pictures on the left represent the simplified observation conditions. The MEP on top of the image is the averaged waveform recorded at baseline condition; below, the MEPs presented were recorded at the initial contact (IC) and toe-off (TO) conditions.

The MEP waveforms obtained in each condition were processed offline, and the peak-to-peak amplitude of the averaged MEP waveform was measured for each participant (Figure 2) using examination software version 3.11.1 (Multi Stim Tracer; Medical Try System Co., Tokyo, Japan). All statistical analyses were performed using SPSS Statistics software version 22 (IBM Co., Armonk, NY, USA). First, to confirm that the alterations of corticospinal excitability did not change throughout the entire experiment, the Wilcoxon signed-rank test was used to compare the baseline and post-observation MEP values. Subsequently, we assessed individual EMG activities, calculated as integrated electromyograms, during a 50-ms period prior to TMS to ensure that no muscle contraction occurred during the TMS measurements, since background EMG activity is known to modulate the size of MEPs induced by TMS [38]. The Friedman test was applied to compare the background EMG activities during the eight stimulus conditions (namely, pre-observation, IC and TO conditions during MJ observation, IC and TO conditions during SJ observation, IC and TO conditions during R-SJ observation, and post-observation). To consider the interindividual variability in TMS-induced motor cortical activity, MEP values in the observation condition were divided by the averaged baseline MEP values and expressed as relative MEP (R-MEP) values. After checking for assumption of sphericity using Mauchly’s test, the two-way repeated measures analysis of variance (ANOVA), with observation condition (MJ, SJ, and R-SJ observation) and stimulus timing (IC and TO) as within-subject factors, was used to examine the effects of observers’ visual attention on corticospinal excitability during the cyclic gait observation. Significant effects were followed by pairwise comparisons using the Bonferroni adjustments. To compare the VAS scores used to evaluate the degree of attention among the three observation conditions, the Friedman test and post hoc comparisons were conducted. Furthermore, the correlation between the R-MEPs in the two stimulus conditions and the VAS score, in which data were obtained from all participants in each observation condition, was analyzed using Spearman’s rank correlation coefficient. The level of statistical significance for all analyses was set at α = 0.05, and effect sizes were reported as partial eta squared (η_p_^2^).

## 3. Results

The Wilcoxon signed-rank test showed no significant differences between the two MEP values measured while observing the same picture of the treadmill before and after the gait observation (*p* = 0.594). This result showed that the excitability in the corticospinal tract was not affected by the short-term gait observation itself and was constant throughout the entire experiment.

The background EMG data expressed as medians (interquartile ranges) are presented in Table 1. There were no significant differences in background EMG activities among the stimulus conditions (*p* = 0.788), indicating that the effects of background activity on changes in MEP could be ruled out in this experiment.

**Figure 3:**
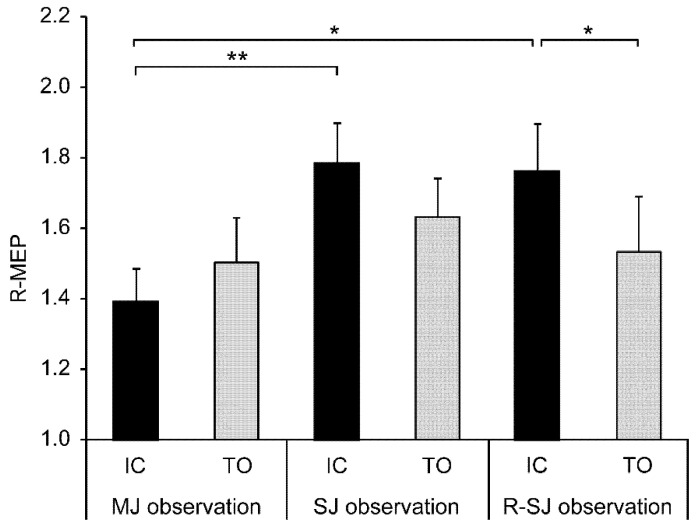
Mean values and standard errors of relative motor evoked potentials (R-MEPs) in two stimulus conditions (IC and TO) during three observation conditions (MJ, SJ, and R-SJ). The MEP values in each condition were divided by the average baseline MEP value and expressed as R-MEP. * *p* < 0.05; ** *p* < 0.01. IC, initial contact; TO, toe-off; MJ, multiple-joint; SJ, single-joint; R-SJ, restricted single-joint.

As a main analysis, the two-way repeated measures ANOVA using R-MEP values revealed a significant observation condition × stimulus timing interaction (F_2,26_ = 4.404, *p* = 0.023, η_p_^2^ = 0.253). However, the main effects of the observation condition (F_2,26_ = 2.982, *p* = 0.068, η_p_^2^ = 0.187) and stimulus timing (F_1,13_ = 2.403, *p* = 0.145, η_p_^2^ = 0.156) were not detected. Post hoc analyses with the Bonferroni correction indicated that the R-MEP values acquired during the SJ (*p* = 0.003) and R-SJ (*p* = 0.026) conditions were significantly higher than those acquired during the MJ condition only in the IC stimulus condition. Moreover, there was a significant difference in the R-MEP value between the IC and TO stimulus conditions only in the R-SJ observation condition (*p* = 0.034) (Figure 3).

**Figure 4:**
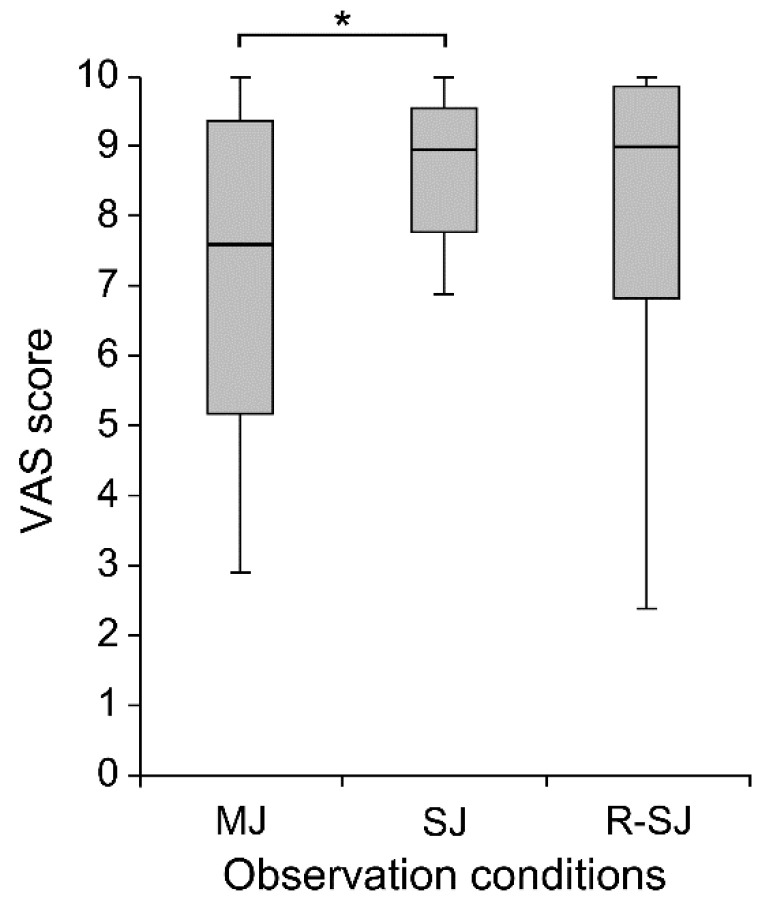
The visual analog scale (VAS) scores evaluating the degree of attention during individual observation conditions (MJ, SJ, and R-SJ) in compliance with the instructions. * *p* < 0.05. MJ, multiple-joint; SJ, single-joint; R-SJ, restricted single-joint.

**Figure 5:**
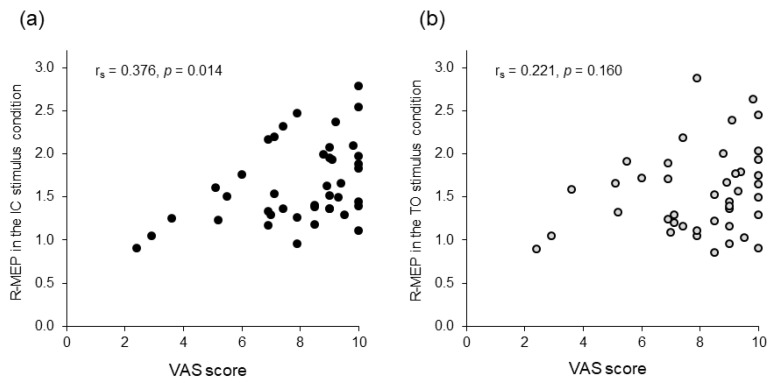
Scattergram representing the relationship between the visual analog scale (VAS) scores and relative motor evoked potentials (R-MEPs). (**a**) Initial contact (IC) stimulus condition. (**b**) Toe-off (TO) stimulus condition. The *x*-axis denotes the degree of attention evaluated using the VAS score. The *y*-axis denotes the R-MEPs as surrogates of changes in corticospinal excitability compared to the baseline condition.

In complementary analyses including the VAS scores, the Friedman test and post hoc multiple comparisons revealed that the VAS score was higher in the SJ than that in the MJ condition (*p* = 0.008); no significant difference was found among the other conditions (Figure 4). Additionally, Spearman’s test showed a significant positive correlation between excitability changes in the corticospinal tract and the VAS score in the IC stimulus condition (r_s_ = 0.376, *p* = 0.014), but not in the TO stimulus condition (r_s_ = 0.221, *p* = 0.160) (Figure 5).

## 4. Discussion

The aim of this study was to explore whether directing visual attention selectively to a body part (a single joint) affects the corticospinal excitability assessed by single-pulse TMS during the observation of whole-body movements, such as the human gait. As we hypothesized, directing the observers’ visual attention to the demonstrator’s ankle joint movement enhanced the excitability of the corticospinal tract to the TA compared to observing the movement of the lower limb freely, without gaze fixation. This facilitated motor cortical response to TMS was a distinctive phenomenon detected only in the IC phase of the gait cycle. This finding was congruent with the results of several previous TMS studies showing that directing visual attention to an acting effector, neighboring region, or task-related object could affect corticospinal excitability, as described for observing finger movements [31], hand or upper limb actions [28,29,39,40,41], and dynamic whole-body actions, such as a golf swing [30].

Regarding the neural correlates of gaze behavior, Maranesi et al. [42] demonstrated that most of the microelectrode-recorded neurons in area F5 discharged more strongly when the macaque monkey looked specifically at the grasping action than when it did not look at it. Similar neural activity associated with this gaze behavior has been found in humans. Corticospinal excitability was reported to be maximized when observers fixated their gaze on a location closest to the main effector or task-relevant features of an action compared with a free-viewing condition without fixing visual attention on a specific location [29,31], whereas corticospinal excitability was diminished when attention was drawn away from the observed action [40]. In this study, the participants were instructed to observe the movements of a walker’s ankle joint in the SJ observation condition or to observe them with limited range of visibility in the R-SJ observation condition; TMS-elicited motor cortical activity during gait observation was recorded from the TA muscle, which is a prime dorsiflexor of the ankle joint. Thus, the fixation of the observer’s gaze on the right ankle joint movement is considered to induce a facilitatory effect on the excitability of the corticospinal tract to the TA compared with the observation of the walker’s lower limb movement without any gaze fixation.

Our supplementary analysis evaluating the degree of attention during gait observation showed that the VAS score of the condition where the participants observed the walker’s ankle joint movement was significantly higher than that of the condition where the participants unrestrictedly observed the walker’s entire lower limb movement. As this result was compatible with the difference in the corticospinal excitability alteration between the MJ and SJ observation conditions, we speculated that the degree of attention and gazing behavior are possible additive factors that activate the observer’s motor system. Functional brain imaging studies have reported the effects of attention on cortical activity during action observation [43,44,45]. Muthukumaraswamy et al. [44] demonstrated that, compared with passive observation conditions, the sensorimotor activity was enhanced in attention-requiring conditions, in which the participants observed finger movement sequences and had to reproduce them or sum the assigned number of presented digits, indicating that paying attention to biological motion stimuli activates the primary sensorimotor cortex. In addition, fixation of the eyes on hand tapping seems to be insufficient to induce μ-suppression on electroencephalography (EEG) (namely, human mirror neuron activity [46]) when attention is diverted and not focused on the action [45]. In summary, the literature findings suggested that the degree of attention (increased or decreased visual attention) to the target action is a crucial factor modulating motor cortical activity during action observation. Hence, as in previous studies [44,45], motor resonance observed in this study appeared to be partially influenced by the extent to which observers concentrated on the visual stimuli.

Interestingly, phase-dependent motor resonance was detected when the observers watched the demonstrator’s ankle joint movements in a restrictive field of vision (R-SJ condition). Despite the comparable situation, in which the observers deliberately and selectively attended to the walker’s foot movements (SJ condition), no significant differences in alterations of corticospinal excitability between the IC and TO stimulus conditions were found during the observation of cyclic gait. This discrepancy in motor resonance between the two observation conditions might reflect the difference in the motor system activity between the two observed actions. Regarding the neural activity controlling the TA muscle during actual walking, the phase-dependent modulation of corticospinal excitability [47,48] and cortical activity [49] was clarified by previous studies using single-pulse TMS and coherence analysis of the coupling between EEG and EMG from the active leg muscle, respectively. However, as no prior studies have addressed the phase-dependent motor cortex drives to the TA muscle during the swing phase, the presence of a more detailed phase modulation during actual walking is still open to discussion. In this regard, although data were obtained using surface EMG; cortical activity was not assessed directly, a study by Byrne et al. [50] clearly showed that the peak amplitude of TA activity during gait was larger at TO than at IC when the treadmill was set at a speed similar to that used in our study. This EMG activity pattern of the TA is completely inverse and inconsistent with the phase modulation of the motor system detected in the R-SJ condition. Additionally, in the peripheral spinal motor system level, the medium-latency responses of the TA were reported to increase at the end of the stance phase relative to those at the end of the swing phase during the mere observation of walking [51]. Taken together, these findings suggested the possibility that the alteration of motor cortical activity shown during the R-SJ condition is not derived from the unalloyed observation of walking.

Donaldson et al. [28] demonstrated that corticospinal excitability during the observation of a tea stir video was positively associated with the number of gaze fixations on the hand and inversely associated with gaze fixations on the object. This result implied that the degree of attention related to gaze fixation affected the magnitude of the TMS-induced motor cortical response. However, in the current study, a positive correlation between the change in the corticospinal excitability and the degree of attention was found only in the IC stimulus condition (not in the TO stimulus condition), indicating that factors other than attention (gaze fixation) could affect corticospinal excitability in the TO stimulus condition. One possible explanation is that the visibility of the observed walker partly affected the excitability in the corticospinal tract [52]. Although most participants denoted a diminished corticospinal excitability in the TO condition relative to the IC condition, some of them (four out of 14) showed an outstanding decline and a relative value < 1.0 in the TO condition. This result indicated that the facilitation effect of gait observation on corticospinal excitability in the TA muscle disappeared or was conspicuously decreased because of the restricted motion visibility. This pattern of motor response to TMS is coincident with muscle-specific resonance: corticospinal excitability in an actual action-related muscle is facilitated during action observation, as reported by several prior TMS studies [12,13,14,15,16,17,18,19,20,21,22]. In the TO stimulus condition, while the ankle joint is observed to be plantarflexed, the TA, which is a dorsiflexor of the ankle joint, becomes activated [34]. This conflict between the observed kinematic motion and the onset of muscle activation seems to affect the way in which the gait video is interpreted; some participants who showed reduced corticospinal excitability in the TO condition probably regarded the gait video in the R-SJ condition not as human walking but as a simple movement of dorsiflexion and plantarflexion of the foot.

However, this study has a limitation in that the exact data of gaze behavior were not measured with an objective method, such as an eye tracker. Although subjective data using VAS showed that the observers were probably able to comply with verbal instruction, it remains unclear to what extent the duration and portion of observers’ gaze fixation affected the increased activity of the motor system, especially during the SJ condition. Further research is required to elucidate the association between gaze fixation on various lower limb movements and the motor resonance of gait-related muscles. This would improve the efficacy of gait observation, as an AOT, in a clinical setting.

## 5. Conclusions

Our findings advanced the comprehension of motor cortical activity during the observation of whole-body movements, such as cyclic human walking. We demonstrated that directing visual attention to a part of the body and focusing on its movement elicited a facilitation effect on the motor system, compared with the free viewing of gait without any gaze fixation. In addition, similar to the difference in cortical activity between walking and voluntary movements, motor resonance can differ between observation of the human gait and that of isolated single-joint movements.

## Figures and Tables

**Table 1:** Median and IQR values of the background EMG activity in the TA muscle during baseline, MJ observation, SJ observation, R-SJ observation, and post-observation conditions.

	Stimulus Conditions							
	Baseline	MJ Observation		SJ Observation		R-SJ Observation		Post- Observation
		IC	TO	IC	TO	IC	TO	
Median	0.119	0.118	0.119	0.118	0.116	0.116	0.118	0.114
(IQR)	(0.112–0.145)	(0.112–0.147)	(0.111–0.147)	(0.111–0.150)	(0.110–0.153)	(0.112–0.147)	(0.113–0.138)	(0.110–0.147)

IQR, interquartile range; EMG, electromyographic; TA, tibialis anterior; MJ, multiple-joint; SJ, single-joint; R-SJ, restricted single-joint; IC, initial contact; TO, toe-off.

## Data Availability

The data that support the findings of this study are available from the corresponding author, T.I., upon reasonable request.
